# MMS Exposure Promotes Increased MtDNA Mutagenesis in the Presence of Replication-Defective Disease-Associated DNA Polymerase γ Variants

**DOI:** 10.1371/journal.pgen.1004748

**Published:** 2014-10-23

**Authors:** Jeffrey D. Stumpf, William C. Copeland

**Affiliations:** Mitochondrial DNA Replication Group, Laboratory of Molecular Genetics, National Institute of Environmental Health Sciences, NIH, DHHS, Research Triangle Park, North Carolina, United States of America; Duke University, United States of America

## Abstract

Mitochondrial DNA (mtDNA) encodes proteins essential for ATP production. Mutant variants of the mtDNA polymerase cause mutagenesis that contributes to aging, genetic diseases, and sensitivity to environmental agents. We interrogated mtDNA replication in *Saccharomyces cerevisiae* strains with disease-associated mutations affecting conserved regions of the mtDNA polymerase, Mip1, in the presence of the wild type Mip1. Mutant frequency arising from mtDNA base substitutions that confer erythromycin resistance and deletions between 21-nucleotide direct repeats was determined. Previously, increased mutagenesis was observed in strains encoding mutant variants that were insufficient to maintain mtDNA and that were not expected to reduce polymerase fidelity or exonuclease proofreading. Increased mutagenesis could be explained by mutant variants stalling the replication fork, thereby predisposing the template DNA to irreparable damage that is bypassed with poor fidelity. This hypothesis suggests that the exogenous base-alkylating agent, methyl methanesulfonate (MMS), would further increase mtDNA mutagenesis. Mitochondrial mutagenesis associated with MMS exposure was increased up to 30-fold in *mip1* mutants containing disease-associated alterations that affect polymerase activity. Disrupting exonuclease activity of mutant variants was not associated with increased spontaneous mutagenesis compared with exonuclease-proficient alleles, suggesting that most or all of the mtDNA was replicated by wild type Mip1. A novel subset of C to G transversions was responsible for about half of the mutants arising after MMS exposure implicating error-prone bypass of methylated cytosines as the predominant mutational mechanism. Exposure to MMS does not disrupt exonuclease activity that suppresses deletions between 21-nucleotide direct repeats, suggesting the MMS-induce mutagenesis is not explained by inactivated exonuclease activity. Further, trace amounts of CdCl_2_ inhibit mtDNA replication but suppresses MMS-induced mutagenesis. These results suggest a novel mechanism wherein mutations that lead to hypermutation by DNA base-damaging agents and associate with mitochondrial disease may contribute to previously unexplained phenomena, such as the wide variation of age of disease onset and acquired mitochondrial toxicities.

## Author Summary

Thousands of mitochondrial DNA (mtDNA) per cell are necessary to maintain energy required for cellular survival in humans. Interfering with the mtDNA polymerase can result in mitochondrial diseases and mitochondrial toxicity. Therefore, it is important to explore new genetic and environmental mechanisms that alter the effectiveness and accuracy of mtDNA replication. This genetic study uses the budding yeast to demonstrate that heterozygous strains harboring disease-associated mutations in the mtDNA polymerase gene in the presence of a wild type copy of the mtDNA polymerase are associated with increased mtDNA point mutagenesis in the presence of methane methylsulfonate, a known base damaging agent. Further observations suggest that the inability of disease-associated variants to replicate mtDNA resulted in increased vulnerability to irreparable base damage that was likely to result in mutations when replicated. Also, this study showed that trace amounts of the environmental contaminant cadmium chloride impairs mtDNA replication but eliminates damage-induced mutagenesis in the remaining functional mitochondria. This interplay between disease-associated variant and wild type polymerase offers new insights on possible disease variation and implicates novel environmental consequences for compound heterozygous patients.

## Introduction

Mitochondrial DNA (mtDNA) maintenance is necessary for the majority of ATP production in eukaryotic cells. The inability to properly replicate mtDNA potentially impacts human health in several ways. The premature aging phenotype of *POLG* exonuclease deficient mice indicates that increased mtDNA mutagenesis can be detrimental [1]–3. Also, mutations in genes encoding the mitochondrial replisome, including DNA polymerase γ (pol γ, encoded by *POLG*), contribute to mitochondrial diseases characterized by mtDNA depletion, deletions, or point mutations [4]–[14]. Additionally, environmental changes can modify mitochondrial biology and potentially impact health. Chain-terminating nucleotide analogs used in anti-viral therapy impair mtDNA replication and can result in mitochondrial toxicity [15]. Antioxidants and exercise have been shown in model systems to improve mitochondrial function and suppress the premature aging phenotype associated with increased point mutations and deletions [3], [14]. Therefore, environmental changes can be important for mitochondrial function, and the mechanisms that cause mtDNA mutations warrant further study.

Currently hundreds of *POLG* mutations have been identified in patients with mitochondrial disease such as Alpers syndrome, progressive external ophthalmoplegia, and ataxia-neuropathy syndrome (mutations listed in http://tools.niehs.nih.gov/polg/) [16]. Pol γ-related mitochondrial diseases display a wide variety of severities. For instance, Alpers syndrome manifests in infants and young children, and these patients rarely live through their first decade of life [17]. Alternatively, patients with progressive external ophthalmoplegia (PEO) and sensory ataxia neuropathy, dysarthria, and ophthalmoparesis (SANDO) often are asymptomatic until>20 years of age [4], [18].

The catalytic subunit of pol γ contains DNA polymerase, 3′-5′ exonuclease, and 5′ dRP lyase activities, with known discrete polymerase and exonuclease domains [19]–[22]. Among the *POLG* mutations associated with mitochondrial disease, many have been characterized biochemically and shown to disrupt polymerase activity [5]–[10], [13], [14], [23]–[27]. *POLG* polymerase variants H932Y, R943H, and Y955C alter dNTP-interacting side chains and are associated with less than 1% polymerase activity [26]. Polymerase variants G848S, T851A, R852C, and R853Q also reduce polymerase activity to <1% of wild type activity; in addition, G848S also exhibits a DNA-binding defect [24]. Although mutagenic effects of point mutations that disrupt exonuclease activity have been well established, disease-associated mutations in the human exonuclease domain surprisingly do not disrupt the exonuclease activity in Mip1 [9], [10]. These disease associated exonuclease mutations have not been studied in the human enzyme.

*S. cerevisiae* has been useful to characterize Pol γ functionality with mutations that alter amino acids within conserved stretches between human *POLG* and yeast *MIP1*, most of which are in the polymerase domain [28], [29]. Mitochondrial functionality and mtDNA point mutagenesis have been determined in various mutants using assays that measure frequency of petite colony formation (ie, lacking mitochondrial function either with [rho^−^] or without mtDNA [rho^0^]) and erythromycin resistance, respectively [30]. For instance, mutations that alter the catalytic aspartates (eg, Asp171 and Asp 230 in yeast) in the exonuclease domain are associated with 1440-fold and 160-fold increases in point mutagenesis [31]–[33] and deletions between direct repeats in mice, respectively [32], [33]; the corresponding increases in yeast are 2000-fold for point mutagenesis [31] and 90-fold for deletions between direct repeats [12], [33]. These increases in mtDNA mutagenesis establish Asp171 and Asp 230 as critical domains for “proofreading” against misinsertions. Surprisingly, disease-associated mutations in the exonuclease domain are associated with only modest increases in mutant frequency, suggesting that exonuclease activity is functionally sufficient to correct misinsertions [9], [10]. Many of the disease-associated mutations to Mip1 eliminated the ability to replicate mtDNA and were associated with petite colony formation, including human variants R807C, R807P, R853W, N864S, G923D, H932Y, K947R, G1076V, R1096C, S1104C, and V1106I and Alpers-associated mutations G848S, T851A, R853Q, D930N, A957P, P1073A, and R1096H [9]. However, several strains that contain variants, including R853Q and Q308H (R656Q and Q264H in yeast) coexpressed with wild type *MIP1* to maintain mtDNA showed significant increases in mutagenesis, and no mechanism has been described for this increase [9].

Environmental agents have also been shown to affect mitochondrial DNA replication both positively and negatively. The presence of antioxidants such as MitoQ and dihydrolipoic acid have been shown to improve mitochondrial function in mutants with disease-associated polymerase domain mutations by salvaging reactive oxygen species [14]. These results suggest an increase of oxidative damage in mtDNA in model systems with defective pol γ, a hypothesis supported by the increased levels of 8-oxo-dG in the mtDNA of a transgenic mouse model that overexpressed the Y955C mutant variant in cardiac tissue [34]. Methyl methanesulfonate (MMS) is an alkylating agent that is associated with increases in mtDNA base damage [35]. Interestingly, in embryonic fibroblasts, 2 mM MMS was associated with persistent mtDNA damage but not with loss of mtDNA or mitochondrial function [36]. In yeast, repair of alkylation damage of mtDNA by MMS involves Apn1 nuclease [37], and Ntg1 [38]. Also, chronic exposure to trace amounts of the known human carcinogen, cadmium chloride, resulted in loss of mitochondrial function [39]. Because cadmium also results in extreme nuclear hypermutability [39], the possibility that cadmium alters mtDNA replication warrants further study.

Base excision repair is active in yeast and human mitochondria and protects cells against alkylation damage [40]. However, lesions on single-stranded DNA are not substrates for base excision repair because there is no complementary strand with which it can reanneal and are therefore highly mutagenic [41]–[43]. The proposed model of asymmetrical mtDNA replication of human mtDNA suggests that single-stranded mtDNA is exposed even in optimal conditions and could be more vulnerable under conditions of decreased replication efficiency [44]. To test whether reduction in mtDNA replication efficiency could leave the cell vulnerable to mutagenic base damage, mtDNA mutagenesis in previously characterized disease-associated mutants were tested in the presence of MMS.

## Results

### Chronic MMS exposure increases mtDNA point mutagenesis in *mip1* mutants

Mutations in *mip1* that result in changes in conserved amino acids previously have been shown to cause defective mtDNA replication and, in some cases, increased mtDNA mutagenesis when coexpressed with wild type *MIP1* [9]. These experiments were performed in haploid heteroallelic strains with intact chromosomal wild type *MIP1* and one of 31 mutant *mip1* alleles on a centromeric plasmid with the endogenous promoter. This study interrogated some of the heteroallelic strains and newly created diploid heterozygotes to determine mtDNA point mutagenesis of the gene encoding the 16S ribosomal subunit that confers resistance to erythromycin and the fraction of cells unable to grow on glycerol which requires mitochondrial function.

**Figure 1:**
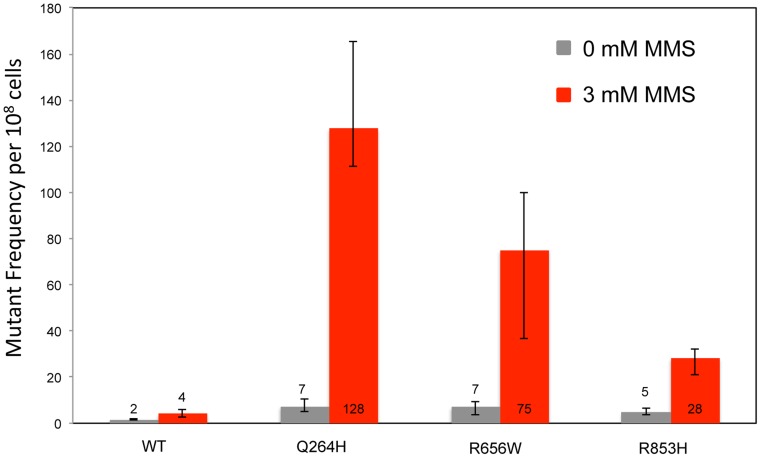
Methyl methanesulfonate exposure increases mtDNA point mutagenesis in heteroallelic strains encoding Mip1 variants. Median frequency (±95% CI) of erythromycin-resistant mutants per 10^8^ rho^+^ cells of haploid strains with wild type *MIP1* encoded in the chromosome and a centromeric plasmid encoding *MIP1* or *mip1* with various disease-associated mutations in the presence (3 mM) or absence (0 mM) of MMS. Mutant frequencies were determined from 40 (for R656W and R853H) and 80 (for MIP1 and Q264H) independent cultures from at least 2 separate experiments. CI, confidence interval; MMS, methyl methanesulfonate; mtDNA, mitochondrial DNA.

To test whether the presence of the catalytically defective mutant variant increases the vulnerability of mtDNA to base damage and mutagenic replication by the wild type polymerase, heteroallelic *S. cerevisiae* strains expressing either Q264H, R656W, R853H, or wild type *MIP1* on a centromeric plasmid and chromosomal wild type *MIP1* were grown in the presence of sublethal concentrations (3 mM) of MMS (see Table 1 for list of all genotypes). None of these mutant proteins are capable of maintaining mtDNA without the presence of wild type Mip1 [9]. MMS exposure caused a modest 2-fold increase in mutagenesis in the wild type control as compared to no exposure to MMS, whereas a greater increase in mtDNA mutagenesis—17-fold, 11-fold, and 6-fold—was observed in strains expressing Q264H, R656W, and R853H, respectively (Figure 1 and Table 1). Absolute mtDNA mutant frequencies after MMS exposures were associated with 30-fold, 18-fold, and 7-fold increases in strains with Q264H, R656W, and R853H mutant variants, respectively, compared with that of the wild type strain. The control strain with wild type *MIP1* on both the centromeric plasmid and the chromosome was associated with only 2.7-fold increase in MMS-induce mutagenesis compared with no MMS exposure.

**Table 1:** List of Pol γ mutations analyzed in this study and summary of results.

Genotype^a^	Human Mutation^b^	Human disease^b^	MMS	CdCl_2_	Median point mutant frequency, Er^R^ colonies per 10^8^ rho^+^ cells (95% CI)	Median deletion mutant frequency, Rho^+^ cells per 10^8^ Arg^+^ cells (95% CI)
**Wild type**						
*MIP1*//*MIP1*			—	—	3.53 (3.19, 3.87)	0 (0, 1.28)
			3 mM	—	7.08 (6.17, 7.99)	1.17 (0, 1.75)
			—	4 µM	9.77 (5.52, 15.18)	
			3 mM	2 µM	18.85 (16.33, 21.35)	
			3 mM	3 µM	16.62 (13.77, 19.57)	
			3 mM	4 µM	27.64 (19.06, 36.22)	
*MIP1/MIP1*			—	—	2.14 (1.82, 2.44)	
			3 mM	—	6.78 (5.73, 7.83)	
			—	4 µM	2.45 (2.22, 2.67)	
			3 mM	2 µM	11.52 (9.73, 13.31)	
			3 mM	3 µM	5.03 (4.33, 5.72)	
			3 mM	4 µM	7.30 (6.16, 8.14)	
**Exonuclease mutations (D171A, E173A)**	**Exo^−^ (D198A, E200A)**	**None identified**				
*MIP1//*D171A-E173A			—	—		39.49 (19.57, 66.67)
			3 mM	—		18.33 (15.56, 42.78)
*MIP1/*D171A-E173A			—	—	62.73 (54.42, 71.14)	
			3 mM	—	42.82 (38.56, 47.08)	
**Q264H**	**Q308H**	**PEO**				
*MIP1//Q264H*			—	—	7.28 (5.11, 10.33)	0.74 (0, 2.21)
			3 mM	—	127.68 (111.34, 165.38)	1.64 (0.82, 4.10)
			—	4 µM	39.72 (36.72, 42.72)	
			3 mM	2 µM	236.14 (190.11, 282.17)	
			3 mM	3 µM	32.95 (27.16, 38.76)	
			3 mM	4 µM	17.36 (13.78, 20.94)	
*MIP1/Q264H*			—	—	17.49 (15.28, 19.70)	
			3 mM	—	46.82 (38.73, 54.93)	
*MIP1/Q264H-* D171A-E173A			—	—	3.90 (2.97, 4.83)	
			3 mM	—	24.23 (18.17, 30.41	
**R656W**	**R853W**	**PEO with ptosis**				
*MIP1//R656W*			—	—	7.07 (3.55, 9.19)	
			3 mM	—	75.07 (36.72, 99.73)	
**R853H**	**R1096H**	**Alpers**		—		
*MIP1//R853H*			—	—	4.61 (3.61, 6.38)	
			3 mM	—	28.11 (21.04, 32.10)	
**R656Q**						
*MIP1/R656Q*			—	—	12.78 (12.02, 13.54)	
			3 mM	—	59.79 (48.38, 71.18)	
**G651S**	**G848S**	**PEO, Alpers, MELAS**				
*MIP1/G651S*			—	—	6.04 (5.36, 6.70)	
			3 mM	—	24.15 (18.59, 29.69)	
*MIP1/G651S-D171A-E173A*			—	—	6.55 (5.10, 8.00)	
			3 mM	—	20.14 (16.57, 23.81)	
*MIP1/R656Q-G651S*			—	—	6.92 (6.22, 7.62)	
			3 mM	—	28.86 (22.52,35.20)	
*MIP1/R656Q-G651S-D171A-E173A*			—	—	6.69 (5.93, 7.43)	
			3 mM	—	47.97 (42.82, 53.12)	
***D891A***	**D1135A**	**None identified**				
*MIP1/D891A*			—	—	46.27 (42.33, 49.21)	
			3 mM	—	216.34 (201.92, 230.74)	
			—	4 µM	40.70 (38.47, 43.23)	
			3 mM	2 µM	158.64 (140.44, 164.84)	
			3 mM	3 µM	22.14 (19.86, 24.42)	
			3 mM	4 µM	53.58 (49.33, 57.83)	

a All strains listed are isogenic in the E134 background (see Materials and Methods).

b The details of the human *POLG* mutations and the associated disease can be found at the Human DNA polymerase γ mutation data base (http://tools.niehs.nih.gov/polg/).

Arg^+^, able to synthesize arginine; CdCl_2_, cadmium chloride; CI, confidence interval; Er, erythromycin; Exo^−^, exonuclease deficient; MELAS, mitochondrial encephalomyopathy, lactic acidosis, and stroke-like episodes; MMS, methyl methanesulfonate; PEO, progressive external ophthalmoplegia.

**Figure 2:**
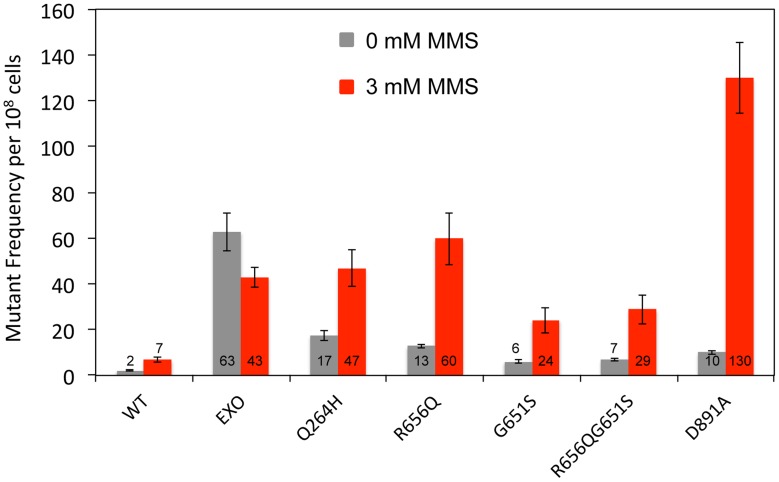
Mutations that disrupted Mip1 polymerase activity caused increased mutant frequency after MMS exposure. Median frequency (±95% CI) of erythromycin-resistant mutants per 10^8^ rho^+^ cells of heterozygous diploid strains containing both *MIP1* and *mip1* with disease-associated mutation, exonuclease disrupting (EXO), or polymerase disrupting mutations in the presence (3 mM) or absence (0 mM) of MMS. Mutant frequencies were determined from at least 20 different independent cultures. CI, confidence interval; MMS, methyl methanesulfonate.

 To avoid the possibility of multicopy expression of the Mip1 variant from the plasmid, heterozygotes were created with mutations that encode Mip1 with defective exonuclease activity [31] or amino acid variants Q264H, R656Q, G651S, or D891A (Table 1). D891A is not associated with mitochondrial disease, but the conserved aspartate in human POLG (Asp1135) is essential for binding catalytic Mg^2+^ in the active site [19]. Alanine substitution of this equivalent residue in other human DNA polymerases has been shown to disrupt binding of the catalytic Mg^2+^, eliminating DNA polymerase activity but not binding to DNA binding [45]. With the exception of Q264H, biochemical characterizations have been reported for the remaining mutant variants [24]. In agreement with previous observations [9], disruption of Mip1 exonuclease activity increased mutagenesis; however, there was no additional increase in MMS-induced mutagenesis (Figure 2 and Table 1). Compared with wild type, Q264H, R656Q, and D891A heterozygotes were associated with 7-fold, 8-fold, and 19-fold increases in absolute MMS-induce mutant frequency. In fact, the resulting increases in mutant frequency were approximately equal to or greater than that of the exonuclease defective variant (Figure 2). Strikingly, MMS exposure of the D891A variant resulted in an approximately 3-fold increase in mutant frequency compared with that of the exonuclease deficient variant. These results demonstrate that catalytically inactive or less active polymerases, whether generated through a site-directed mutation or a disease-associated mutation, participate in a mechanism that causes MMS-induced mutations.

Mip1 variants R656Q and G651S are homologous to human disease variant R853Q and G848S, respectively, which both exhibit ≤1% catalytic activity [24]. However, G848S uniquely displayed an approximately 5-fold reduction in DNA binding [24]. Interestingly, Mip1 G651S variant resulted in fewer MMS-induced mutations compared with the other polymerase variants, suggesting that DNA binding may be important for the mechanism. To test this hypothesis, a R656Q/G651S double mutant was created, and MMS-induced mutagenesis was compared with each single mutant. Mutagenesis after exposure to MMS in the double mutant was indistinguishable from the G651S variant and lower than in a single R656Q mutant (Figure 2), suggesting that DNA binding is an important component of the R656Q mutator effect.

### Mutant Mip1 variants do not replicate mtDNA

**Figure 3:**
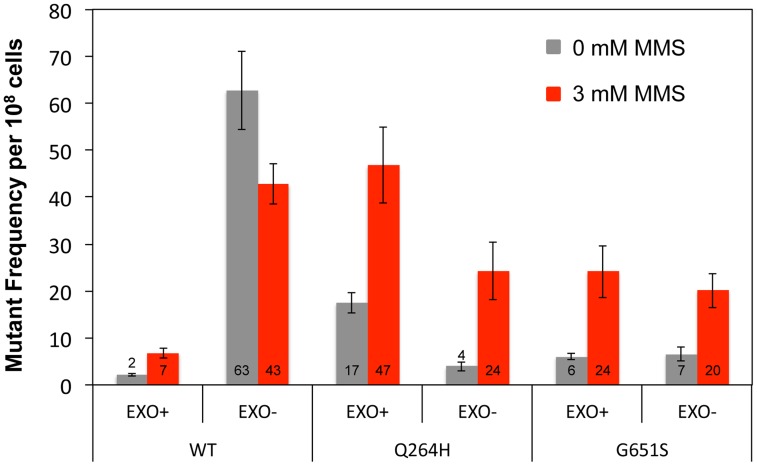
Q264H and G651S Mip1 variants were not significantly involved in mtDNA replication. Median frequency (±95% CI) of erythromycin-resistant mutants per 10^8^ rho^+^ cells of heterozygous diploid strains containing both a wild type *MIP1* allele and a *mip1* allele with disease-associated mutation with or without exonuclease disrupting mutations *in cis* in the presence (3 mM) or absence (0 mM) of MMS. Mutant frequencies were determined from at least 20 different independent cultures. CI, confidence interval, MMS, methyl methanesulfonate; mtDNA, mitochondrial DNA.

To test whether Mip1 mutant variants participate in the bulk of mtDNA replication, mutagenesis was measured in heterozygous diploids that had one wild type *MIP1* allele and one allele containing mutations that disrupt exonuclease activity and encode the Q264H and G651S variants *in cis*. Unlike exonuclease-deficient Mip1 without other mutations, eliminating exonuclease activity did not increase mutagenesis in either the Q264H or G651S variant, with or without MMS exposure (Figure 3 and Table 1). In fact, mutation frequency unexpectedly decreased when the exo^−^ and Q264H were *in cis*. These results suggest minimal contributions of Q264H and G651S mutant variants to mtDNA replication.

### MMS exposure is associated with increased C:G→G:C transversions

**Figure 4:**
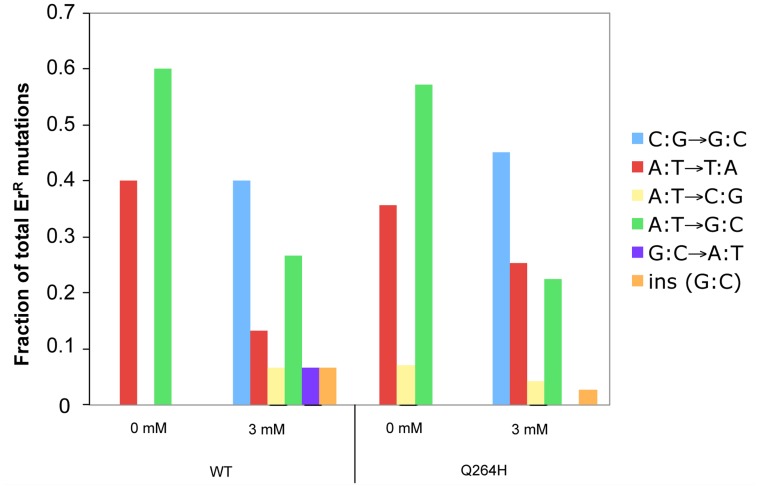
Methyl methanesulfonate exposure resulted in C:G→G:C transversions in mtDNA. Fractions of different base substitutions in heterozygous strains with a wild type (WT) *MIP1* allele and a mutant *mip1* encoding the Q264H variant in the presence (3 mM) or absence (0 mM) of MMS. The following number of mutants was sequenced for each group: Wt (0 mM MMS), 5; Wt (3 mM MMS), 15; Q264H (0 mM MMS), 28; Q264H (0 mM MMS), 71. *P*-values for the C to G mutations were calculated using a one-tailed Fisher's Exact test. The *p*-value for the 0.46 fraction of G to C mutations with the Q264H and 3 mM MMS as compared to no MMS was <0.0001. The *p*-value of the 0.40 fraction of G to C mutations for the WT comparing 3 mM MMS with no MMS was 0.17 and <0.0001 using our WT no MMS data and the WT data from Kalifa and Sia [46], respectively. MMS, methyl methanesulfonate; mtDNA, mitochondrial DNA; Wt, wild type.

Resistance to erythromycin is conferred by one mutation at any of the following nucleotides: 1950 (G to T or G to A), 1951 (A to T, A to G, or A to C), 1952 (A to T or A to G), 3993 (C to G), or an insertion of G between nucleotide 1949 and 1950 of the 21S rRNA gene (Gen Bank accession number L36885) [31], [46], [47]. To determine if there was a change in the spectrum of mutations associated with MMS exposure, PCR fragments containing nucleotides 1797–1995 and 3895–4107 of the 16S ribosomal subunit gene from erythromycin resistant mutants were sequenced. Only one mutant was taken from each original culture to ensure that each mutation represented a separate event. In the absence of MMS, this and prior studies [46]–[48] demonstrate that A:T→G:C and A:T→T:A were the most frequent mutations (Figure 4 and Table S1). In this and previous studies, C to G mutations were not detected in wild type strains and were detected in only 5% of Δ*rev1* strains [46]. Interestingly, we found that exposure to MMS was associated with a significant change in mutational spectrum, wherein the most common mutation was C:G→G:C transversions in both the wild type strain (40%) and Q264H heteroallelic strain (45%). G:C→A:T was the only mutation other than C:G mutation detected, but it was only detected in the wild type strain and very low levels (7%). These results suggest that cytosine or guanine is especially sensitive to methylation by MMS, leading mostly to misincorporated cytosines or guanines, similar to previous studies [43].

### MMS exposure does not increase MtDNA mutations by reducing exonuclease activity

**Figure 5:**
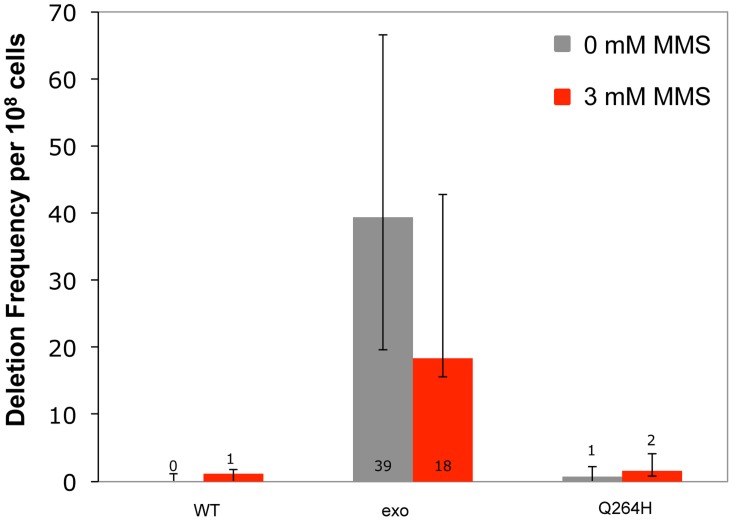
Methyl methanesulfonate exposure has no significant effect on deletion mutations between 21mer repeats. Median frequency (±95% CI) of deletions between 96-mer direct repeats per 10^8^ Arg^+^ cells of heteroallelic strains containing a centromeric plasmid encoding *MIP1* or *mip1* with an exonuclease-disrupting mutation or a mutation encoding the Q264H variant in the presence (3 mM) or absence (0 mM) of methyl methanesulfonate. Mutant frequencies were determined from at least 20 different independent cultures. CI, confidence interval; MMS, methyl methanesulfonate.

Increased mutagenesis can arise by disrupting exonuclease activity and/or increasing the frequency of nucleotide misincorporation events. Previous work demonstrated that mitochondrial deletions between direct repeats of 21 nucleotides were rare events that were suppressed by exonuclease activity [12]. To test whether MMS promotes deletion formation, haploid deletion reporter strains that were heteroallelic for wild type, exonuclease-deficient, or Q264H variants of Mip1 were used to measure frequency of deletions between 21 nucleotide direct repeats that flank an *ARG8* insertion in the mitochondrial genome. Frequency of deletions between direct repeats was increased (40-fold) in the strain with the exonuclease-deficient Mip1 variant but was not significantly different in the strain with the Q264H variant compared with wild type (Figure 5 and Table 1). MMS had no significant effect on deletion formation in any of the three strains, suggesting that the effect of MMS is specific to point mutations.

### Trace amounts of cadmium chloride reduces mtDNA content and suppresses mtDNA mutagenesis

**Figure 6:**
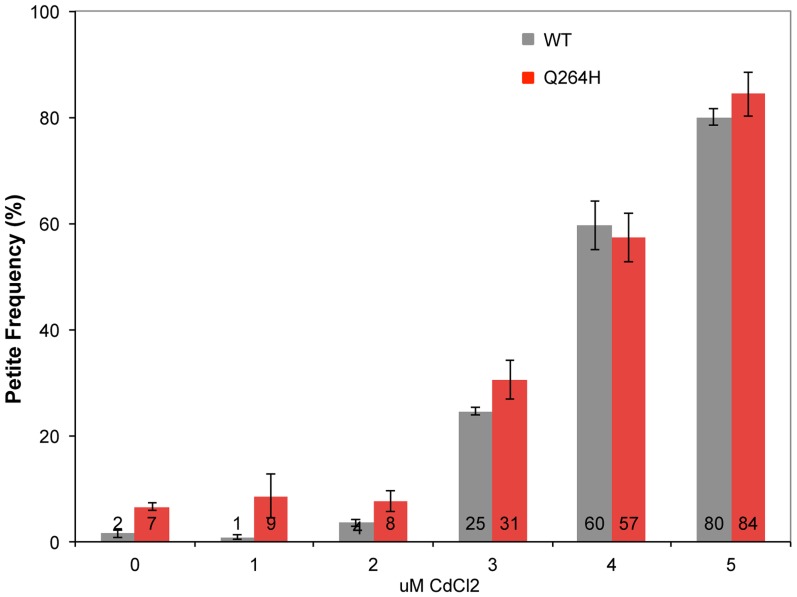
Cadmium chloride increases petite formation and decreases mtDNA replication. Petite colony formation of heteroallelic strains containing a centromeric plasmid encoding *MIP1* or *mip1* with the mutation encoding the Q264H variant after exposure to different concentrations of cadmium chloride. mtDNA, mitochondrial DNA; nDNA, nuclear DNA.

Mutations associated with MMS exposure occur in strains with mutants that affect mtDNA replication, suggesting that the mechanism requires suboptimal mtDNA replication. Therefore, it is possible that an environmental agent that reduces mtDNA replication may also be associated with MMS-induced mutagenesis in wild type cells. This was tested by treating wild type and mutant *mip1* strains with CdCl_2_. Exposure to 3 µM CdCl_2_ was associated with increased petite formation frequency of about 30% with stepwise increases at 4 and 5 µM of about 60% and 80%, respectively (Figure 6) in both wild type and Q264H mutants. Exposure to 5 µM CdCl_2_ was associated with 3.6-fold reduction in mtDNA among rho^+^ cells, from 30.6±4.0 copies per cell without CdCl_2_ exposure to 8.5±0.9 copies per cell with 5 µM CdCl_2_, suggesting that trace amounts of CdCl_2_ are associated with mtDNA depletion.

**Figure 7:**
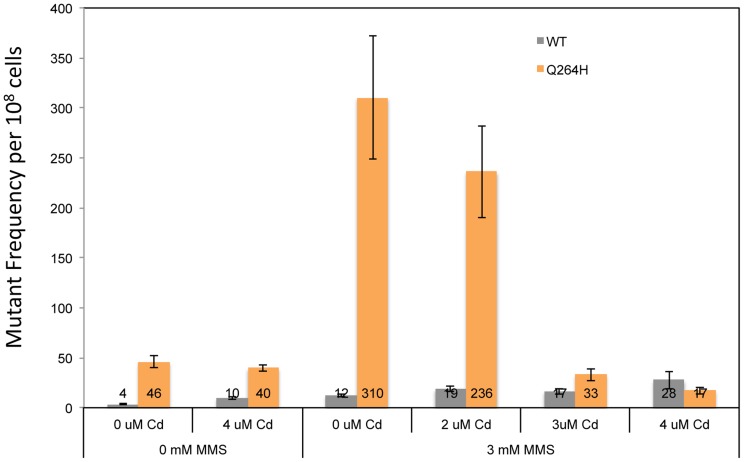
Exposure to CdCl_2_ suppressed the MMS-induced increase in MtDNA mutant frequency. Median frequency (±95% CI) of erythromycin-resistant mutants per 10^8^ rho^+^ cells of heterozygous diploid strains containing both a wild type *MIP1* allele and a *mip1* allele encoding the Q264H variant in the presence or absence of methyl methanesulfonate (0 or 3 mM) or CdCl_2_ (0, 2, 3, or 4 µM). Mutant frequencies were determined from at least 20 different independent cultures. CI, confidence interval; MMS, methyl methanesulfonate, mtDNA, mitochondrial DNA.

Mitochondrial DNA mutagenesis was assayed in homozygous wild type diploid cells and Q264H heterozygotes to test if MMS-induced mtDNA mutagenesis occurs with CdCl_2_. Exposure to 4 µM CdCl_2_ had no effect on mtDNA mutagenesis in either the wild type or Q264H mutant strains (Figure 7). Therefore, the mutagenic effect of CdCl_2_ is specific to nuclear DNA [39]. Exposure to both MMS and CdCl_2_ resulted in no increase in mtDNA mutagenesis. However, the mutagenic effect of MMS observed in the heteroallelic Q264H strain was completely suppressed by 3 µM or 4 µM CdCl_2_. The lack of mtDNA mutagenicity and the suppression of the MMS-induced mtDNA mutagenesis by trace amounts of CdCl_2_ were recapitulated in heterozygotes expressing the D891A mutant variant (Table 1). Although reduced efficiency of mtDNA replication by a mutant variant is associated with MMS-induced mutagenesis, these results suggest that processes that reduce mtDNA replication suppress MMS mutagenesis.

## Discussion

This study demonstrates a novel mechanism of MMS-induced mtDNA point mutagenesis, mostly C:G→G:C transversions, in heterozygous strains with a disease-associated mutation that disrupts polymerase activity. The frequency of the mutagenesis appeared to be modulated by the activity of the mutant variant in that the possible DNA binding defect observed in the human homologue to G651S reduced MMS-induced mutagenesis. This study showed that alleles with added exonuclease defect were not associated with increased mutagenesis (with or without MMS exposure), suggesting that the mutant variant replicated little or none of the mtDNA that remained and was propagated in the cell. Finally, chronic exposure to trace amounts (3–5 µM) of CdCl_2_ resulted in the inability to replicate mtDNA, which surprisingly did not increase but instead suppressed MMS-induced mutagenesis. These results are the first to support a mechanism to understand the interplay between polymerases in heterozygous cells and reveal a novel pathway for environmentally-induced mtDNA mutagenesis.

There are few known pathways that increase mtDNA mutations in yeast or humans. Mutations that disrupt the exonuclease activity of the mtDNA polymerase have been shown to cause increased mtDNA mutagenesis. One study used a reversion assay in yeast to identify several mitochondrial mutators including *pos5*, a gene that encodes an NADPH kinase [49]. Other genes associated with increased mtDNA mutagenesis—such as *hap2*, *fen1*, and *ntg1*—have been identified, but their effect on mtDNA mutagenesis has been modest or requiring long incubation times [49]–[51]. Even base damaging agents such as H_2_O_2_ and MMS are associated with modest increases in mtDNA but only in strains without crucial repair pathways [52].

Previously, disease-associated mutations were shown to increase mtDNA mutagenesis, but these mutations also led to the inability to maintain functional mitochondria because of mtDNA depletion [9]. The increase in mutant frequency in some strains was evident in this study in the unexposed controls (Figures 1 and 2). Therefore, it was difficult to ascertain how mutant polymerases that in some cases were suggested to have little or no activity could significantly increase mtDNA mutagenesis. Exposing the heteroallelic strains to sublethal doses of MMS resulted in up to 30-fold increases in mtDNA mutant frequency (Figure 1). Interestingly, repeating the experiment in heterozygous strains recapitulated the MMS-induced increase, albeit at a lower frequency (Figure 2). The heteroallelic strain contains the mutant *mip1* and its endogenous promoter on a centromeric plasmid that has been previously shown to have 1–2 copies of the gene per cell [13]. It is possible that MMS-induced mutagenesis is sensitive to differences in the number of Mip1 mutant copies. The fact that the heteroallelic strains are haploid whereas the heterozygotes are diploid could suggest that increased copy number of other replication proteins may alter the MMS-induced mutagenesis phenotype, although there is probably no difference in the protein concentration relative to the genome copy number.

MMS-induced mutagenesis was shown in several disease-associated mutants and the polymerase defective mutant but not the exonuclease-deficient mutant. Although Q264H is an exonuclease domain disease-associated variant, it was previously shown to be detrimental to mtDNA replication. Mutations that result in the Mip1 R656Q and G651S variants are homologous to the mutations in the human pol γ thumb domain that were biochemically characterized to have <1% polymerase activity including a 5-fold reduction in DNA binding affinity in the G651S homologue [24]. Interestingly, G651S is associated with reduced mtDNA mutant frequency and suppression of R656Q when the two mutations are *in cis*. These results suggest that lower DNA binding affinity impedes the mechanism of MMS-induced mutagenesis. Gly651 is in a stretch of amino acids conserved between humans and yeast, making it likely that Gly651 is also involved in DNA binding. However, future studies will be necessary to show that G651S does not possess other characteristics (eg, decreased stability) that impair MMS-induced mutagenesis.

Biochemical evidence suggesting that some disease-associated mutant variants were impaired for mtDNA replication was further supported by the observation that the mutant variants by themselves could not maintain mtDNA [9]. However, it has been unclear to what extent these polymerases function in the cell. It is well known that one of the catalytic aspartates (Asp891 in Mip1) is necessary for mtDNA replication; therefore, D891A is unable to catalyze the polymerase reaction. Interestingly, the heterozygous strain with D891A was associated with the largest increase in MMS mutagenesis, approximately 3-fold more than the exonuclease-deficient strain. Because the generation of mutations requires DNA replication, this result indicates that the wild type polymerase, which is normally accurate, becomes more likely to incorporate the wrong nucleotide in the D891A strain upon MMS exposure. In the case of the disease-associated mutations, it is possible that the mutant variants contribute to the incorporation of the incorrect nucleotide. However, removal of exonuclease activity *in cis* with Q264H and G651S showed no increase in mutation frequency regardless of MMS exposure and even showed an unexpected reduction of mutagenesis in the Q264H strain. Considering that the mutant frequency in the Q264H/exo^−^ strain was similar to the G651S mutant frequency, this study cannot discount the possibility that the combination of the two mutations may have similar characteristics to G651S (eg, lower DNA binding affinity). Regardless, these results suggest that the mutant polymerase is not contributing directly to the mutations that drive the mutant frequency. It is possible that the mutant variants replicate mtDNA molecules that may be selected against or are not propagated, possibly because of incomplete replication.

Resistance to erythromycin is associated with a limited mutational spectrum that rarely includes C:G→G:C transversions [46]–[48]. Interestingly, exposure to MMS dramatically changed the mutation spectrum such that 40–45% of the mutations were C:G→G:C transversions. These mutations could either arise from a cytosine incorporated opposite a cytosine or a guanine incorporated opposite a guanine. Previously, MMS exposure of artificially formed or random ssDNA in yeast was associated with increased frequency of all 3 kinds of cytosine substitutions, C→T, C→G and C→A [42], [43]. The mutation spectra and strand bias suggested that N3-methyl cytosine is the prominent mutagenic lesion caused by MMS lesion in the yeast nuclear ssDNA [42], [43]. Therefore, it is possible that C:G→G:C transversions that predominate after MMS exposure result from cytosine incorporation opposite of a methylated cytosine.

**Figure 8:**
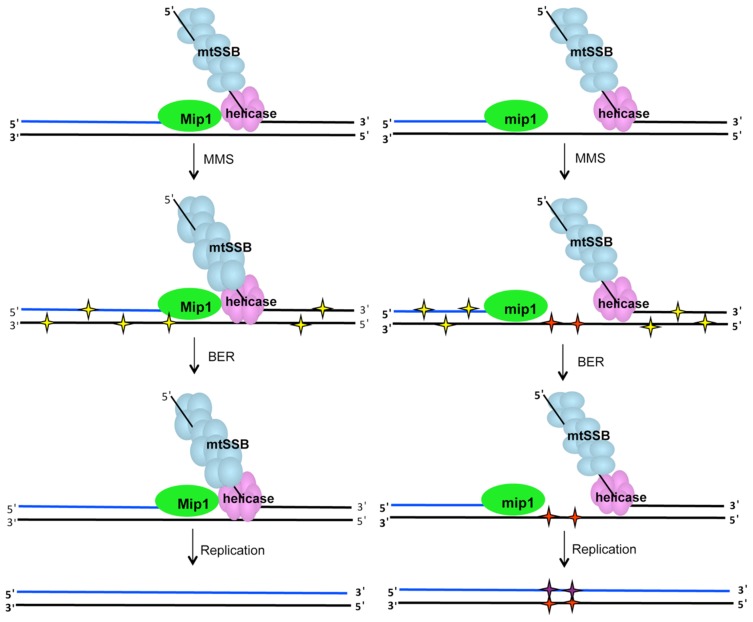
MMS-induced mutagenesis in Mip1 variants may be associated with unrepaired ssDNA damage. Under normal circumstances (left), single-stranded gaps at the replication fork are limited because of Mip1 processivity. However, reduced catalytic activity of the Mip1 disease variants (right scheme) results in the greater likelihood of stalling, which could produce long tracks of ssDNA if the helicase proceeds normally. When exposed to MMS, potentially mutagenic lesions in dsDNA (yellow stars) as well as N3-methylated cytosines in ssDNA (red stars) are produced. Although, BER is expected to correct the base damage in dsDNA, ssDNA lesions are not a BER substrate and are therefore unrepaired. Replication of the lesion is likely to result in incorporation of a cytosine opposite the N3-methylated cytosine to make the C:G→G:C mutation. BER, base excision repair; dsDNA, double-stranded DNA; MMS, methyl methanesulfonate; ssDNA, single-stranded DNA.

These combined results support a model (Figure 8) wherein disease-associated mutant polymerase variants bind to and temporarily stall mtDNA replication, an event that could result in ssDNA intermediates (eg, because of polymerase-helicase uncoupling). Although mtDNA base excision repair would normally repair most damaged bases in dsDNA, there are no known repair systems that act on ssDNA in yeast. In the absence of MMS, the endogenous sources of DNA damage (eg, oxidative damage) impart a small but significant amount of DNA damage, whereas MMS magnifies the damage. Either the mtDNA continues to be stalled and degraded (an event that would not yield a mutant colony) or polymerase switching might occur, allowing the wild type polymerase access to the replication fork. In some cases the polymerase would incorporate the incorrect nucleotide leading to the development of a mutation. This model suggests that the mutant polymerase would stably bind DNA but be unable to replicate mtDNA efficiently.

Recently it was shown that the exonuclease domain suppresses mtDNA deletions between 21-mer direct repeats up to 160-fold [12]. One hypothesis could be that MMS also inhibits exonuclease activity which would lead to an increase in mutagenesis. This hypothesis was unlikely because MMS exhibited a modest effect on the diploid wild type strain. This study showed that MMS did not increase the frequency of mtDNA deletion mutants in wild type or Q264H background suggesting that the mechanism that caused MMS-induced point mutations is different than that of mtDNA deletion formation. Furthermore, MMS does not alter the exonuclease activity involved in suppression of mtDNA deletion formation.

A previous report showed that exposure to trace amounts of CdCl_2_ was associated not only with suppression of nuclear mismatch repair but also increase in petite colony formation frequency [39]. This study recapitulates this finding and further shows that CdCl_2_ does not significantly affect mtDNA mutant frequency. These results indicate that either there is no efficient mismatch repair system in yeast mtDNA or the amount of CdCl_2_ needed to observe a defect in mismatch repair is similar to the amount that is associated with a high frequency of dysfunctional mitochondrial. The only mismatch repair homologue associated with yeast mitochondria is Msh1, and it has been proposed to play a role in base excision repair [48]. This study also showed that mtDNA content was reduced in rho^+^ cells exposed to CdCl_2_ suggesting that cadmium negatively affects mtDNA replication or maintenance. We tested whether the effect of inhibiting mtDNA replication could mimic the effect of a disease-associated mutation by increasing MMS-induced mutagenesis. Unexpectedly, 3 and 4 µM CdCl_2_ did not promote MMS-induced mutagenesis in wild type cells. It is possible that CdCl_2_ does not stall replication but rather affects another replication-related process, such as replication initiation or termination. Even more unexpectedly, CdCl_2_ suppressed MMS-induced mutagenesis associated with Q264H. It should be noted that mutant frequency is determined among only rho^+^ cells so increased petite colony formation frequency from CdCl_2_ exposure does not explain the suppression of mutant frequency. One possible explanation is that the presence of CdCl_2_ selects against the maintenance of mtDNA molecules that are damaged or stalled either through some direct inhibition of the enzymes involved or as a result of a cellular response to mtDNA stress. Another possibility is that damaged mtDNA is more sensitive to CdCl_2_-induced inhibition of mtDNA replication, and the replication of these mtDNA molecules are not completed or maintained during the growth of the colony. Therefore, the various combinations of environmental exposures and genetics may be a useful tool to understand different pathways that occur in response to DNA damage.

This study shows a novel gene-environment interaction which greatly increases mtDNA mutagenesis and supports a polymerase-switching mechanism that has not been described in mtDNA replication. The interpretations of this study are limited in humans because there are key differences in yeast mtDNA maintenance compared with mammalian mtDNA (eg, lower mtDNA copy number and high frequency of recombination). Also, the study assumes that the mutant frequency (ie, mutants per culture) is indicative of the mutation frequency (mutations per mitochondrial division). True mutation frequencies would require information on mtDNA kinetics and mitochondrial dynamics that is currently unavailable. However, with the advent of high-throughput genome sequencing, similar studies in mtDNA mutagenesis will be possible in a model system that more closely mimics human mtDNA. Interestingly, this interaction involves heterozygotes containing mutations which are normally associated with disease [53]. It is interesting to consider that if a similar mechanism occurs in mammalian mtDNA, people who are heterozygous for a disease-associated mutation could be sensitive to environmental exposures that would impair mtDNA replication and promote symptoms involved in mitochondrial toxicity or disease.

## Materials and Methods

### Media and growth conditions

*S. cerevisiae* strains were grown at 30°C in YP (yeast extract 1%, peptone 2%) with 2% glucose or glycerol as carbon sources or synthetic complete media. *Escherichia coli* strains were grown in standard LB media at 37°C. When appropriate, gentamicin and ampicillin were added to YPD (0.2 mg/ml) and LB (0.1 mg/ml), respectively.

### Plasmid constructions

The plasmid, pFL39, which contains *MIP1* on a centromeric plasmid was previously described [31]. Site-directed mutagenesis of plasmid-encoded *MIP1* was performed using the QuikChange Site-Directed Mutagenesis Kit (Invitrogen) as described previously. The construction of plasmids containing *ACT1, COX1*, and *COX2* fragments, used for mtDNA quantitation was previously described.

### Strain constructions

All *S. cerevisiae* strains were derived from E134 (MATα *ade5-1 his7-2 lys2-A14 leu2-3,112 ura3-52 trp1-289*) [54] and its MATa isogenic strain, YH747. Heteroallelic *mip*1 strains were made by transforming pFL39-*MIP1* or a mutant derivative into E134 by selecting for TRP. Strains that measure deletions between direct repeats were created by transforming *TRP1*-containing plasmids PFL39 [12] containing wild type *MIP1* or *mip1* encoding an exonuclease-deficient mutant variant (*mip1-exo* [Het]; JSY114) into *trp1*::G418 NPY75 (JSY77) [12]. Chromosomal *mip1* mutations were created in haploid E134 with wild type *MIP1* using a PCR-based *delitto perfetto* method as previously described. All strains were checked phenotypically for the absence of the CORE casette used in *delitto perfetto* (ie, screening for Ura^−^ and gentamicin sensitive cells) and were sequenced to confirm the presence of the mutation and the absence of undesired nearby mutations. The *mip1* haploid mutant strains were mated with the isogenic E134 with mating type a.

### Point and deletion mutant frequency measurements

Resistance to erythromycin is conferred by one of several missense mutations in the 21S rRNA gene in mitochondrial DNA [46], [47], [55]. Yeast strains were replica plated onto YPD plates with or without 1–5 mM MMS or 1–5 µM CdCl_2_ and grown at 30C for 2 days. In most cases 3 mM MMS was the highest exposure that allowed growth and was used for the experiments. After plating on CdCl_2_, increased chromosomal mutagenesis was confirmed qualitatively by the presence of colonies on media lacking lysine. From these plates, 20–40 independent colonies from each strain were used to inoculate into 4 ml of synthetic media without tryptophan (heteroallelic strains) or YPD (heterozygous strains), and these cultures were incubated to saturation (for 2 days) at 30°C. The cells were plated on YPEG (1.7% ethanol, 2% glycerol) with 4 g/L erythromycin. A small aliquot of 5–10 cultures were used to titer the number of rho^+^ cells by plating 10^−5^ dilutions on YPG. Erythromycin resistant colonies were counted after 6 days of incubation at 30°C. The mutant frequency was the median number of erythromycin colonies per 10^8^ rho^+^ cells plated. To determine the spectrum of erythromycin-resistant mutations, DNA was extracted from one mutant per culture and used for as a template in a PCR reaction to amplify two regions, approximately 200 nucleotides flanking nucleotides 1950 (using 5′-GAGGTCCCGCATGAATGACG and 5′-CGATCTATCTAATTACAGTAAAGC) and 3993 (using 5′-CTATGTTTGCCACCTCGATGTC and 5′-CAATAGATACACCATGGGTTGATTC). The resulting amplified DNA was the template for the sequence reactions.

To measure deletions between direct repeats, all strains were replica plated onto YPD with or without MMS exposure as described above. Independent cultures were grown from at least 20 colonies at 30°C in YP (yeast extract 1%, peptone 2%) with 2% glucose and adenine for 2 days. Appropriate dilutions of samples from the saturated cultures were plated on synthetic complete media lacking arginine to determine total number of cells with mtDNA. The cultures were plated onto YP with 2% glycerol and deletion mutants were counted after 4 days. The mutant frequency was determined as the median number of mutant colonies per 10^8^ Arg^+^ cells. In all mutagenesis experiments, 95% confidence levels were determined using the method of the median.

### Petite frequency measurements

Petite frequencies are the frequency of rho^−^ cells (petites) in the total population. Rho^−^cells are devoid of mitochondrial functions but are not necessarily devoid of mtDNA (rho^0^). To determine petite frequency in heteroallelic strains, at least 12 fresh transformants per strain were diluted in water and between 200–1000 colonies were plated onto YPD. For monoallelic strains, rho^+^ cells derived from several tetrad dissections were single colony purified on YPD and then assayed for petite frequency. Cells were incubated at 30°C for 2 days. Rho^+^ cells were identified either by the accumulation of red pigment as a result of mutations in the *ade* biosynthetic pathway or by the inability to grow on YPG. Using either or both method, at least 300 colonies per plate were counted, and petite colonies were identified. Frequencies were determined for each plate, and the median number of the frequencies was calculated. 95% confidence levels were determined by using the method of the median (52). For monoallelic strains determined to be 100% petite, no rho^+^ cells could be isolated from cells derived from 10 different haploid spores.

### Mitochondrial DNA quantitation

Mitochondrial DNA copy number was quantified relative to nuclear DNA copy number using real time PCR. Primers and probes designed to specifically amplify within the mitochondrial-encoded *COX1* gene and the nuclear-encoded *ACT1* gene. Real time PCR reactions using Taqman Universal PCR Master Mix (Applied Biosystems) were performed at 40 cycles of 95 for 30 sec and 50 degrees for 30 sec. Known concentrations of plasmid molecules containing *COX1*and *ACT1* were quantified as a positive control [9] and real time PCR was performed on 7 different dilutions to determine a logarithmic equation of a curve (R^2^ values>0.98) that represents numbers of molecules as a function of the critical threshold of every reaction. Every reaction was done in triplicate, and three replicates were tested for each experimental condition. Data represent the average ratio (± SEM) of the number of *COX1* molecules to the number of *ACT1* molecules.

## Supporting Information

Table S1 MMS mutation spectrum from DNA sequence analysis. Sequence analysis from erythromycin clones of the five known positions in the yeast 21S mtDNA gene that confer this resistance (position 1950 (G to T or G to A), 1951 (A to T, A to G, or A to C), 1952 (A to T or A to G), 3993 (C to G), or an insertion of G between nucleotide 1949 and 1950 in the 21S rRNA mtDNA gene (Gen Bank accession number L36885).
(DOCX)
