# Supplementary material for: MMS Exposure Promotes Increased MtDNA Mutagenesis in the Presence of Replication-Defective Disease-Associated DNA Polymerase γ Variants
Source: PLoS Genet. 2014 Oct 23;10(10):e1004748. doi: 10.1371/journal.pgen.1004748 (PMC4207668; doi:10.1371/journal.pgen.1004748)
Supplement: Table S1 — MMS mutation spectrum from DNA sequence analysis. Sequence analysis from erythromycin clones of the five known positions in the yeast 21S mtDNA gene that confer this resistance (position 1950 (G to T or G to A), 1951 (A to T, A to G, or A to C), 1952 (A to T or A to G), 3993 (C to G), or an insertion of G between nucleotide 1949 and 1950 in the 21S rRNA mtDNA gene (Gen Bank accession number L36885). (DOCX) [file pgen.1004748.s001.docx]

| **Table S1**. MMS mutation spectrum from DNA sequence analysis  Sequence analysis from erythromycin clones of the five known positions in the yeast 21S mtDNA gene that confer this resistance (position 1950 (G to T or G to A), 1951 (A to T, A to G, or A to C), 1952 (A to T or A to G), 3993 (C to G), or an insertion of G between nucleotide 1949 and 1950 in the 21S rRNA mtDNA gene (Gen Bank accession number L36885). | | | | |
| --- | --- | --- | --- | --- |
| Nucleotide change | Wt/wt 0mM MMS | Wt/wt  3mM MMS | Wt/Q264H  0mM MMS | Wt/Q264H 3mM MMS |
| C:G→G:C | 0 | 6 | 0 | 32 |
| A:T→T:A | 2 | 2 | 10 | 18 |
| A:T→C:G | 0 | 1 | 2 | 3 |
| A:T→G:C | 3 | 4 | 16 | 16 |
| G:C→A:T | 0 | 1 | 0 | 0 |
| +1 G:C | 0 | 1 | 0 | 2 |
| total | 5 | 15 | 28 | 71 |
